# Looking for an Objective Parameter to Identify Early Vocal Dysfunctions in Healthy Perceived Singers

**DOI:** 10.1007/s12070-023-03726-0

**Published:** 2023-04-11

**Authors:** Lucia Longo, Diletta Angeletti, Silvia Parrinello, Armando Cilfone, Claudia Giliberti, Raffaele Mariconte

**Affiliations:** 1 Dipartimento Organi di Senso, Università Sapienza Roma, Rome, Italy; 2 INAIL Dipartimento Innovazioni Tecnologiche e Sicurezza Degli Impianti, Prodotti ed Insediamenti Antropici, 00143 Rome, Italy

**Keywords:** I-low, SVHI, Professional singer, MDVP, DSI, Vocal dysfunction

## Abstract

The finding of minimal laryngeal dysfunctions in professional voice users is essential to prevent the onset of organic vocal pathologies. The purpose of this study is to identify an objective parameter that supports the phoniatric evaluation in detecting minimal laryngeal dysfunctions in singers. 54 professional and non-professional singers have been evaluated with laryngostroboscopy, Multi-Dimensional Voice Program (MDVP), Dysphonia Severity Index (DSI), maximum phonation time (TMF), minimum intensity of sound emission (I-min), maximum frequency (F-max), voice handicap index (VHI), singing voice handicap index (SVHI), manual phonogram and audiometric examination. The SVHI of all the “healthy” singers was on average 23.7 ± 22.5, while that of the “dysfunctional” 20.9 ± 18. No statistically significant difference was found between the SVHI scores of the total of healthy singers compared to the scores of the dysfunctional ones on the VSL (*p* = 0.6). The between-group comparison of the means of individual parameter values of DSI, TMF, F-max, Jitter, Shimmer, NHR, and SPI was not statistically significant (respectively *p* = 0.315, 0.2, 0.18, 0.09, 0.2, 0.08, 0.3). The only parameter analyzed that was statistically significant was the I-min (*p* < 0.05). SVHI is a valid instrument for the evaluation after a therapy but in our experience, it is not useful in distinguishing healthy from dysfunctional patients. The minimum intensity of sound emission measured with the sound level meter (I-low2) resulted a reliable parameter to identify minimal laryngeal dysfunctions and a useful tool in supporting the phoniatric diagnostic-therapeutic process in singers.

## Introduction

Teachers, actors, lawyers, call center operators, tour guides, singers are some professional figures whose vocal organs are subjected to excessive strain. These voice professionals can be considered as a group of workers at risk for developing Work Related Voice Disorders (WRVD) [1].

WRVD represents any form of vocal change, directly related to the use of the voice, that could reduce, impair or impede the worker's performance, having an impact even on life quality [2, 3].

Changes in the fundamental frequency, intensity and vocal timbre, hoarseness, up to the onset of pathologies as laryngitis, polyps, nodules, etc. can occur.

The development of WRVD for singers is multifactorial, being associated with several factors that can directly or indirectly trigger or worsen the worker's vocal impairment [3].

Non-occupational risks include factors such as gender, age, respiratory allergies, upper respiratory tract diseases, hormonal influences, medications, alcohol, smoking and coffee abuse, poor hydration, inappropriate diets with increased risk of pharyngolaryngeal reflux, and non-professional activities with high vocal demand.

The professional risk factors [3] include extensive use of the voice, poor rest, stress, anxiety, sudden changes in temperature, inadequate ambient ventilation, exposure to chemical substances irritating the upper respiratory tract (solvents, fumes, etc.), postural alterations [4], presence of dust and/or cigarette smoke, etc.

Among the professional figures considered at risk for the onset of WRVD, teachers and singers are extensively studied in the literature, since they tend to report voice disturbances more frequently than the general population [2].

A recent meta-analysis indicates a higher mean prevalence of self-reported vocal problems in singers than in the general population [5].

Perkner et al. [6] found a significant increase in voice impairment and vocal disability compared to controls in three groups of singers (opera, musical theater and modern singers) [7].

Among the most recurrent pathologies, gastroesophageal reflux is particularly present among opera singers, due to the prolonged stress on the diaphragm. The results of a study on 351 professional singers and 578 controls show that in this group, the occurrence of the pathology is almost double compared to the population [8].

A 2009 study shows that voice professionals report a prevalence of heartburn, regurgitation and hoarseness, statistically higher than a control group and significantly correlated with the length of working life in the sector [9].

The assesment of a patient with a voice problem is multidimentional and includes the use of clinician instruments (fibrolaryngoscope, aerodynamic and acoustic test, etc.). In particular, laryngostroboscopy allows to identify anatomical and functional vocal cords abnormalities by viewing the appearance and movement of the vocal fold in a slow-motion video format.

Vocal health can be assessed instrumentally, but singers have effective and sensitive assessment tools to identify potential vocal problems themselves, before any laryngeal disease occurs. This result can be achieved by using a self perceived questionnaire that has been proven to effectively record the patients’ experience of their voice disorders [10, 11].

Studies have reported that the Voice Handicap Index (VHI) questionnaire [12] represents an important tool for specialists to check preliminarly how perceived voice impairment can affect social, emotional and professional comfort [13].

Based on the VHI score, subjects can be classified according to 3 degrees of severity of dysphonia: Mild (0–30): minimum amount of handicap; Moderate (31–60): often seen in patients with nodules, polyps, or cysts vowels; Severe (61–120): often observed in patients with vocal cord paralysis or severe vocal fold and scarring.

The Singing Voice Handicap Index (SVHI) is a validated questionnaire to evaluate the function and quality of the voice, specific for singers [11].

This study aims to identify an objective parameter that supports the phoniatric evaluation to discriminate and diagnose early laryngeal dysfunctions in singers who minimize and neglect vocal problems, revealed but instrumental analysis.

## Material and Methods

The study was conducted at the Phoniatric Unit of the Otolaryngology Clinic in “Policlinico Umberto I”, Rome from september 2019 to june 2021, as part of a research activity with the Santa Cecilia Conservatory of Rome and the diocesan choir of Rome.

The study was designed in accordance with the Declaration of Helsinki (‘World Medical Association Declaration of Helsinki’, 2013). Each participant was informed about the research protocol, the long-term use of data and the right to withdraw from the study. Informed consent was provided by all participants.

All the singers of the Santa Cecilia Conservatory of Rome and the diocesan choir of Rome were invited to a free screening at the clinics and, with prior informed consent, were subjected to clinical and instrumental evaluation.

The singers who responded to the invitation and voluntarily accepted to be part of the research were 54, of which 6 professional singers, 28 choristers, 20 vocal students. The group consisted of 35 women and 19 men, aged 20 to 53, with an average age of 29,6 years, with 80% under 35.

After an initial interview with an explanation of the research protocol and collection of informed consent, the patients underwent a medical history questionnaire, self-administration of the SVHI questionnaire, fibrolaryngoscopy, stroboscopy, manual phonogram, audiometric examination and voice recording.

To evaluate voice quality, a recording of vocal samples was performed, with comfortable emission of the vowel /a/ held at constant pitch and intensity for a few seconds, analyzed with the multi-parameter software Multi-Dimensional Voice Program (MDVP).

The acoustic analysis made it possible to obtain the maximum phonation time, identification of the minimum intensity of sound emission (I-low) and maximum frequency (F-high).

The exclusion criteria were ongoing acute inflammatory pharyngolaryngeal, ongoing nasosinusal diseases and vocal cords neoformations on stroboscopic analysis.

Moreover, the stroboscopic findings in which dysfunctional dysphonias were present, were taken into consideration (for example hyperkinesia, hypokinesia, posterior triangular deficit, hyperkinesis of the false cords, etc.) excluding the organic dysphonias in which a suffering of the chordal tissue is assumed.

According with these assumptions, of the 54 singers enrolled, 5 were excluded, of which 1 professional singer suffering from right vocal cord edema, 1 lyric singing student suffering from two angiomas of the left vocal cord, 3 choristers with pre-contact, nodular attitude and edema on the left vocal cord.

The patients finally included in the study were 49 singers, (24 opera and 25 pop music singers), aged between 19 and 53 years, 30 women with an average age of 29 years and 19 men with an average age of 29 years. The mean age of professional singers was 39 years, while 28,2 was the mean age for the others.

All statistical procedures were performed using t Student's parametric statistical test.

### Laryngostroboscopy

All singers underwent fibrolaringoscopy with videolaryngostroboscopy (VLS) with a 70° rigid laryngoscope (Hopkins, 8700 CKA, Karl Storz, Germany) and flexible nasopharingoscope, (XION EF-N 3.4 mm, Inventis SRL Padova Italy), a reliable method to detect lesion size rating, the anteroposterior supraglottic compression and the glottal closure. The instrumental examinations were carried out by a team of 2 otorhinolaryngologists, specialists with 30 years of experience.

The vocal cords morphology, leveling, length and motility were examined as well as the supraglottic structures attitude and glottal closure.

### Multi-dimensional Voice Program (MDVP)

Calculation of the vocal parameters was carried out with the MDVP system (Mode) 5105, Version 3.1.4 © 2000–2006 Kay PENTAX) according with the Sifel protocol on dysphonia:

Vocal /a/ held, for at least 4 s, without sound interruptions, silent environment (< 30 dB of background noise); microphone 20 cm from the lips; conversation voice intensity, between 55 and 65 dB on average; constancy of intensity and frequency; direct digital recording of the entire vocalization.

Only the central three seconds of the vocalization were considered, the attack and the emission extinction have been eliminated.

Of the 33 measurable parameters with this program, we mainly considered Jitter%, Shimmer%, Noise to Harmonic Ratio (NHR) and Soft Phonation Index (SPI).

In addition, the following parameters were analyzed:

Maximum phonation time (TMF): calculated with a stopwatch asking the subject to emit a /a/ comfortable in frequency and intensity as long as possible after a deep inspiration. The best of the three performances is taken into consideration.

Maximum frequency (F-max): two measurements were taken, F-max1 and F-max2. The F-max1 was extrapolated from the phonogram, as the Sifel protocol suggests, indicating the maximum frequency reached by the singer during the rehearsal.

The F-max2 was measured through the frequency counter of the stroboscope with a frequency detector placed on the patient's neck which records the vocal emission frequency, while the subject emits a /a/ spoken (almost shouted) at the maximum possible frequency (trying not to sing/turn the sound).

Minimum intensity of sound emission in dBA (I-min) has been calculated with two methods, obtaining an I-low1 and an I-low2.

The I-low1 was extrapolated, as the F-max1, from the phonogram taking the minimum intensity.

The I-low2 was measured with the following method: Vocal /a/ held, for at least 4 s, without interruption of sonority at the minimum emissive intensity, silent environment (< 30 dB background noise), class I sound level meter (brand 01 dB model SOLO) at 20 cm from the lips, angled 45°, 3 training tests, direct recording of the entire time history of the issue vocal with 10 samples per second of equivalent sound level weighted A (LegA), extraction from the acquired time history of a number of samples sufficient in an almost constant emissive tract in order to obtain a minimum A-weighted equivalent sound level.

These last three parameters (TMF, F-max and I-min) together with the Jitter% allowed to calculate the Dysphonia Severity Index (DSI).

By means of a Fisher linear discriminative analysis the following formula is defined:

$${\text{DSI}} = 0.13 \times {\text{TMF}} + 0.0053 \times {\text{F - }}\max - 0.26 \times {\text{I - }}\min - 1.18 \times {\text{Jitter}}\% + 12.4$$

### Voice Handicap Index (VHI) and Singing Voice Handicap Index (SVHI)

The VHI was not modified, and it was administered according with the Sifel protocol. The SVHI was slightly modified in the translation by a professional translator to better render some expressions which could lead to confusion.

### Phonogram

The phonogram was done manually by a musician and by an acoustic engineer inside a silent booth with a background noise of less than 30 dB.

The notes were indicated by the musician through a digital piano and analyzed by the acoustic engineer through a class I sound level meter (brand 01 dB model SOLO); the authors decided not to use a frequency analyzer because the musician was able to understand if the note produced was adequately in tune or waning/rising.

The notes C, E, G and A within the range of each singer and at the extremes of the range were analyzed proceeding by semitones. The analysis began with the middle notes with medium frequency and then it proceeded towards the low notes and then the high ones.

The singer was positioned 30 cm away from the sound level meter and emitted the note indicated by the musician at the minimum and maximum possible intensity.

After sampling the entire extension, the individual traces recorded were analyzed separately to report the minimum and maximum intensity values on the Cartesian graph.

### Audiometry

Each patient underwent a tonal audiometry in headphones, to exclude hearing loss.

## Results

Out of the total sample, 24 are professionals, 5 graduated singers (4 females and 1 male) and 19 singing students from Conservatory Santa Cecilia in Rome (12 females and 7 males) and 25 choristers of the choir of the diocese of Rome (14 females and 11 males).

The medical records showed 6 patients already operated on at the laryngeal level (4 for nodules and 2 for edema), 21 patients with gastroesophageal reflux (2 with evident clinical signs, 19 with occasional symptoms).

Through stroboscopic evaluation we found 29 healthy singers and 20 dysfunctional singers. For the dysfunctional ones, 7 with posterior triangular deficit, 4 with signs of laryngopharyngeal reflux, 2 with hyperkinesis of the supraglottic structures, 2 with mild hyperemia and hyperkinesis of the supraglottic structures, 1 with posterior triangular deficit and hyperkinesis of the false cords, 2 with posterior triangular deficit and 2 with central closure deficit.

The use of the SVHI aims to investigate the correlation between the level of self-perceived disability, measured precisely through the Singing Voice Handicap Index (SVHI) and the presence of an effective dysfunctional laryngopathy or an unacknowledged organic laryngopathy, verifiable through videostrobolaryngoscopy (VSL) in a group of singers.

The SVHI score of the total sample ranges from a minimum of 0 to a maximum of 89/144, with a mean of 22.6 ± 20.6.

Professional singers scored a mean SVHI of 14.4 ± 18.2; singing students 18.7 ± 16.2; chorist singers 25.3 ± 23.7. Opera singers achieved an SVHI mean of 18.6 ± 16.5; after VSL, 13 of them were found to be normal for laryngeal morphology and motility (mean SVHI: 21.6), while 11 were classified as dysfunctional (mean SVHI: 15). Pop singers scored a mean SVHI of 26.4 ± 23.7; of these, 16 were normal on VSL (mean SVHI: 25.4), while 9 showed dysfunction (mean SVHI: 29.1).

The SVHI of all the "healthy" singers was on average 23.7 ± 22.5, while that of the "dysfunctional" 20.9 ± 18.

No statistically significant difference was found between the SVHI scores of the total of healthy singers compared to the scores of the dysfunctional ones on the VSL (*p* = 0.6).

The finding of an SVHI score inversely proportional to the level of vocal experience underscores the role of an established technique in limiting self-perceived discomfort. It is conceivable the development of virtuous compensation mechanisms in opera singers constantly exposed to dysfunctionality from vocal hyperfunction, such as not to affect the quality of their performance.

The identification of minimal laryngeal dysfunctions in subjects who use the voice as the main professional tool is essential to prevent the onset of organic vocal pathologies. With the aim of identifying an objective parameter that supports the phoniatric evaluation for the identification of minimal laryngeal dysfunctions in the singer, an acoustical analysis of the voice was performed.

Healthy singers showed mean DSI of 4.4 ± 2.1, TMF of 18.2 ± 5.9 s, F-max of 642.7 ± 274 Hz, Jitter of 0.389 ± 0.211%, Shimmer of 2.513 ± 0.994%, Noise Harmonic Ratio (NHR) of 0.125 ± 0.014, Soft Phonation Index (SPI) of 5.672 ± 3.398, I-min of 51.1 ± 4.9 dB.

Singers with minimal laryngeal dysfunction had a mean DSI of 3.8 ± 2, TMF of 16.4 ± 5 s, F-max of 748.6 ± 265.2 Hz, Jitter of 0.463 ± 0.260%, of Shimmer of 3.086 ± 1.382%, of NHR of 0.134 ± 0.02, of SPI of 4.79 ± 2.578, of I-min of 54.4 ± 5.3 dB.

The between-group comparison of the means of individual parameter values of DSI, TMF, F-max, Jitter, Shimmer, NHR, and SPI was not statistically significant (respectively *p* = 0.315; *p* = 0.2; *p* = 0.18; *p* = 0.09; *p* = 0.2; *p* = 0.08; *p* = 0.3).

The only parameter analyzed that was statistically significant was the I-min (*p* < 0.05).

Singers with minimal laryngeal dysfunction develop an unconscious increase in subglottic pressure and an increase in laryngeal tensoadductor forces with high vocal cost and difficulty producing a low pitched sound compared to healthy singers.

The I-min in this group represents a reliable parameter for identifying the slightest dysfunctions at the laryngeal level, representing a useful objective support tool in the singer's phoniatric diagnostic-therapeutic process.

Audiometry resulted normal in all the patients.

The results are presented in Tables 1 and 2.

**Table 1:** Results analysis

Singers	Min.	Max.	Average	SD	Min.	Max.	Average	SD
	VHI				SVHI			
Healthy	0	57	15.44	14.92	0	89	23.72	22.54
Dysfunctional	2	57	14.75	12.606	2	64	20.85	17.98
	Jitter %				Shimmer %			
Healthy	0.192	1.1	0.389	0.211	0.089	4.519	2.513	0.994
Dysfunctional	0.181	1.018	0.463	0.260	1.482	6.548	3.086	1.382
	NHR				SPI			
Healthy	0.102	0.153	0.125	0.014	1.043	15.094	5.672	3.398
Dysfunctional	0.109	0.182	0.134	0.02	1.806	10.796	4.79	2.578
	F-max1 (Hz)				F-max2 (Hz)			
Healthy	262	1048	642.7	274.09	228	505	358.4	82.61
Dysfunctional	330	1184	748.6	265.29	140	780	388.2	185.5
	I-low1 (dB)				I-low2 (dB)			
Healthy	42	63	51.86	6.170	43.4	63	51.1	4.959
Dysfunctional	44	63	55.24	6.168	42.1	65.2	54.46	5.308
	MTF (s)				DSI			
Healthy	10.88	32	18.23	5.925	− 0.888	7.365	4.43	2.186
Dysfunctional	8	26.11	16.46	5.032	− 0.019	6.946	3.803	2.041

**Table 2:** *P* value resulted from the comparison between the parameters of healthy and dysfunctional singers

	VHI	SVHI	Jitter %	Shimmer %	NHR	SPI
*p* value	0.86	0.6	0.09	0.2	0.08	0.3

	F-max1 (Hz)	F-max2 (Hz)	I-low1 (dB)	I-low2 (dB)	MTF	DSI
*p* value	0.18	0.4	0.065	0.02	0.2	0.315

## Discussion

According to Cohen et al. [14] SVHI is a reliable and valid tool to measuring the impact of vocal problems on the singer [15]. As in other studies in the literature, we found unknow laryngeal pathologies in singers [16]. These pathologies were unknown probably because these do not affect singing or because the singers learned to adapt them. Based on our laryngostroboscopies we identified 29 healthy and 20 dysfunctional singers suffering from adductor deficits, hyperkinesis of the supraglottic structures and signs of laryngopharyngeal reflux.

Singers with an organic pathology were excluded from the study, based on SVHI we obtained 13 pathological results, of which only 4 were classified as dysfunctional by laryngostroboscopy. We also compared the scores of the 3 areas of investigation in healthy and dysfunctional singers, and we noted that higher scores have been obtained for healthy singers.

Moreover, we found that the psychological components were equivalent to those specific to the vocal production.

Although we believe in a holistic evaluation of the singer's voice, considering health as a multidimensional concept, which incorporates the physical, mental, and social state of being, it is appropriate to evaluate all these areas separately, as the separated scores of VHI, with the aim to identify more accurately what is the problem triggering the disody [17–20].

Currently, all the questions are evaluated as a single result preventing correct analysis of the 3 areas of investigation.

Moreover, the questions regarding the voice emission characteristics are only 10 out of 36 and are not specific. Since singers are particularly attentive to mild and precocious symptoms, adding more specific items focused exclusively on the technique of sung vocal production, we could have a preliminary test to the laryngostroboscopic instrumental examination and a valid tool in the diagnostic-therapeutic process [21, 22].

Although the SVHI may be adequate to assessing the perceived impact of known vocal problems, it is unable to accurately assess, or correlate with personal perception, the presence of pathology misunderstood in healthy singers, as also found by Castelblanco et al. [23–25].

SVHI is a mainly psychological instrument valid for the evaluation after a therapy, but it does not allow us to identify problems and to distinguish healthy and dysfunctional singers.

Since the SVHI is not useful to identify pre-pathological situations in the singer, we analyzed the values obtained from the MDVP, more specifically the Jitter%, the Shimmer%, the NHR, the SPI, and Soft Phonation Index, to understand if one of these parameters could act as a predictive index [26–30]. No significant differences emerged on these four parameters. The low SPI value indicates a greater richness of harmonics and high frequency formants and the presence of an efficient singing formant with a good vocal projection attitude, therefore we expected lower values in the singers. Instead, according to Rodrigues [31] et al. and Felippe et al. [32], we obtained a well-known statistically significant difference regarding the NHR values between men and women (*p* = 0.0004). It may be related to the fact that men use a voice characterized by less glottic closure and this favors vocal production with fewer harmonics and greater amount of noise [33]. This parameter, however, does not allow us to distinguish between healthy and dysfunctional singers. Therefore, some performance parameters such as the maximum phonation time, the maximum emitted frequency and the minimum intensity of sound emission have been analyzed to calculate the DSI. The maximum phonation time resulted significantly different only between men and women (*p* = 0.02) [34]. Contrary to Schmidt et al. results that found a prolonged MPT in the group of singers, we didn’t find other differences [27].

An only gender difference has been highlighted on the maximum frequencies. Regarding this parameter, we drawn two values for each singer, one extracted from the phonogram and the other one measured with the stroboscope, but we have seen how the value obtained from the phonographical examination is more reliable.

About the minimum intensity, according to the authors' experience, any artifacts, or irregularities in DSI calculation can occur especially if this parameter is not carefully evaluated. Given that according to Sifel protocol this method needs to be tested by various operators for its validation®, we have also calculated it using another method (I-low2 values). The first value (I-low1) resulted by phonogram and the second one (I-low2) was measured with sound level meter. By comparing the values of the minimum sound emission intensity between healthy subjects and those of dysfunctional subjects, we obtained a statistically significant difference only regarding the values obtained through our measurement method (*p* = 0.02), and for this reason, considering them more reliable, we used these values for the calculation of the DSI. Looking at the values of the two categories, dysfunctional subjects (I-low = 54.46) have a significantly higher values than healthy subjects (I-low = 51.1). This could be explained by the fact that singers with minimal laryngeal dysfunction, such as minimal glottic incompetence or hyperkinesis, need more subglottic pressure to counteract the greater muscle force necessary for adduction and subsequent chordal vibration and therefore produce a sound of greater intensity than healthy singers. In clinical practice, therefore, the evaluation of I-low2 could be very useful for identifying clinically healthy but dysfunctional patients. Considering the ease of measurement of I-low2, which does not necessarily require a specialist’s evaluation, it could be a very useful and inexpensive tool. Moreover, the three performance parameters (MTF, F-max1, I-min2), together with the Jitter%, allowed us to calculate the Dysphonia Severity Index (DSI). DSI was developed by Wuyts et al. [35], with the aim to developing an index objectively and quantitatively correlated to the quality of the perceived voice. According to Hakkesteegt et al. [36], DSI should be interpreted as a measure of vocal function/performance that is not necessarily related to perceived vocal quality or measured. However, we did not obtain statistically significant differences in DSI.

## Conclusion

The identification of minimal laryngeal dysfunctions in singer is essential to prevent the onset of organic vocal pathologies. The multiparametric voice evaluation performed in professional and non-professional singers showed that the minimum intensity of sound emission measured with the sound level meter (I-low2) is a useful tool identify laryngeal dysfunctions in healthy subjects.

In our opinion I-low2 is a reliable and minimally invasive measurement that can be performed also by the speech therapist during the therapy. Also, the SVHI is a valid instrument for the evaluation after a therapy but based on our experience it is not useful in distinguishing healthy from dysfunctional patients. Further studies are needed to obtain a standardized method of measuring the minimum sound emission intensity usable to predict laryngeal dysfunction in healthy subjects.

## References

[1] Devadas U, Hegde M, Maruthy S (2019). Prevalence and risk factors of self-reported voice problems among yakshagana artists. J Voice.

[2] Pestana PM, Vaz-Freitas S, Manso MC (2017). Prevalence of voice disorders in singers: systematic review and meta-analysis. J Voice.

[3] Przysiezny PE, Przysiezny LT (2015). Work-related voice disorder. Braz J Otorhinolaryngol.

[4] Longo L, Di Stadio A, Ralli M, Marinucci I, Ruoppolo G, Dipietro L, De Vincentiis M, Greco A (2019). Voice parameter changes in professional musician-singers singing with and without an instrument: the effect of body posture. Folia Phoniatr Logop.

[5] Baird BJ, Mokhtari TE, Sung CK, Erickson-DiRenzo E (2020). A preliminary study of vocal health among collegiate a cappella singers. J Voice..

[6] Perkner JJ, Fennelly KP, Balkisson R (1999). Self-reported voice problems among three groups of professional singers. J Voice.

[7] Williams NR (2003). Occupational groups at risk of voice disorders: a review of the literature. Occup Med (Lond).

[8] Cammarota G, Masala G, Cianci R, Palli D, Capaccio P, Schindler A, Cuoco L, Galli J, Ierardi E, Cannizzaro O, Caselli M, Dore MP, Bendinelli B, Gasbarrini G (2007). Reflux symptoms in professional opera choristers. Gastroenterology.

[9] Pregun I, Bakucz T, Banai J, Molnár L, Pavlik G, Altorjay I, Orosz P, Csernay L, Tulassay Z, Herszényi L (2009). Gastroesophageal reflux disease: work-related disease?. Dig Dis.

[10] Ugolino AC, Oliveira G, Behlau M (2012). Disfonia na percepção do clínico e do paciente. J Soc Bras Fonoaudiol.

[11] Baracca G, Cantarella G, Forti S, Pignataro L, Fussi F (2014). Validation of the italian version of the singing voice handicap index. Eur Arch Otorhinolaryngol.

[12] Nanjundeswaran C, Jacobson BH, Gartner-Schmidt J, Verdolini AK (2015). Vocal fatigue index (VFI): development and validation. J Voice.

[13] Tafiadis D, Chronopoulos SK, Helidoni ME, Kosma EI, Voniati L, Papadopoulos P, Murry T, Ziavra N, Velegrakis GA (2019). Checking for voice disorders without clinical intervention: the Greek and global VHI thresholds for voice disordered patients. Sci Rep.

[14] Cohen SM, Jacobson BH, Garrett CG (2007). Creation and validation of the singing voice handicap index. Ann Otol Rhinol Laryngol.

[15] Meerschman I, D’haeseleer E, Cammu H (2022). Voice quality of choir singers and the effect of a performance on the voice. J Voice.

[16] Arffa RE, Krishna P, Gartner-Schmidt J (2012). Normative values for the voice handicap index-10. J Voice.

[17] Rosen CA, Lee AS, Osborne J (2004). Development and validation of the voice handicap index-10. Laryngoscope.

[18] Montojo J, Garmendia G, Cobeta I (2006). Comparación entre los resultados del fonetograma manual y el fonetograma automático [Comparison of the results obtained through manual and automatic phonetogram]. Acta Otorrinolaringol Esp.

[19] Sataloff RT (1981). Professional singers: the science and art of clinical care. Am J Otolaryngol.

[20] Schindler A, Ottaviani F, Mozzanica F (2010). Cross-cultural adaptation and validation of the Voice Handicap Index into Italian. J Voice.

[21] Damsté PH (1970). The phonetogram. Pract Otorhinolaryngol (Basel).

[22] Rosen CA (2005). Stroboscopy as a research instrument: development of a perceptual evaluation tool. Laryngoscope.

[23] Castelblanco L, Habib M, Stein DJ (2014). Singing voice handicap and videostrobolaryngoscopy in healthy professional singers. J Voice.

[24] Elias ME, Sataloff RT, Rosen DC (1997). Normal strobovideolaryngoscopy: variability in healthy singers. J Voice.

[25] Sobol M, Sielska-Badurek EM, Osuch-Wójcikiewicz E (2020). Normative values for singing voice handicap index -systematic review and meta-analysis. Braz J Otorhinolaryngol..

[26] Nicastri M, Chiarella G, Gallo LV (2004). Multidimensional Voice Program (MDVP) and amplitude variation parameters in euphonic adult subjects. Normative Study Acta Otorhinolaryngol Ital.

[27] Schmidt P, Klingholz F, Martin F (1988). Influence of pitch, voice sound pressure, and vowel quality on the maximum phonation time. J Voice.

[28] Schutte HK, Seidner W (1983). Recommendation by the Union of European Phoniatricians (UEP): standardizing voice area measurement/phonetography. Folia Phoniatr (Basel).

[29] Zhang Z (2015). Regulation of glottal closure and airflow in a three-dimensional phonation model: implications for vocal intensity control. J Acoust Soc Am.

[30] McCulloch TM, Van Daele D, Ciucci MR (2011). Otolaryngology head and neck surgery: an integrative view of the larynx. Head Neck..

[31] Rodrigues S, Behlau M, Pontes P (1994). Proporcao harmonico-ruido: valores para individuos adultos brasileiros. Acta AWHO.

[32] Naufel de Felippe AC, Grillo MH, Grechi TH (2006). Standardization of acoustic measures for normal voice patterns. Braz J Otorhinolaryngol..

[33] Van Lierde KM, De Bodt M, Dhaeseleer E (2010). The treatment of muscle tension dysphonia: a comparison of two treatment techniques by means of an objective multiparameter approach. J Voice.

[34] Heylen LG, Wuyts FL, Mertens FW (1996). Phonetography in voice diagnoses. Acta Otorhinolaryngol Belg.

[35] Wuyts FL, De Bodt MS, Molenberghs G (2000). The dysphonia severity index: an objective measure of vocal quality based on a multiparameter approach. J Speech Lang Hear Res.

[36] Hakkesteegt MM, Brocaar MP, Wieringa MH (2008). The relationship between perceptual evaluation and objective multiparametric evaluation of dysphonia severity. J Voice.

